# Serum hepcidin: indication of its role as an “acute phase” marker in febrile children

**DOI:** 10.1186/1824-7288-39-25

**Published:** 2013-04-25

**Authors:** Lydia Kossiva, Alexandra Soldatou, Dimitrios I Gourgiotis, Lamprini Stamati, Charalampos Tsentidis

**Affiliations:** 1 Second Department of Pediatrics ‘P&A Kyriakou’ Children’s Hospital, Medical School, Athens University, Athens, Greece; 2 Laboratory of Clinical Biochemistry-Molecular Diagnostics, Second Department of Pediatrics ‘P&A Kyriakou’ Children’s Hospital, Medical School, Athens University, Athens, Greece; 3 Second Department of Pediatrics ‘P&A Kyriakou’ Children’s Hospital, Athens University, Medical School, Thivon & Levadias str, 11527 Goudi, Athens, Greece

**Keywords:** Hepcidin, Acute infection, Children, Marker

## Abstract

**Background:**

Hepcidin is classified as a type II acute phase protein; its production is a component of the innate immune response to infections.

**Objective:**

To evaluate the alterations of serum hepcidin in children during and following an acute febrile infection.

**Materials and methods:**

22 children with fever of acute onset (< 6 hours) admitted to the 2^nd^ Department of Pediatrics-University of Athens. Based on clinical and laboratory findings our sample formed two groups: the viral infection group (13 children) and the bacterial infection group (9 children). Hepcidin, ferritin and serum iron measurements were performed in all subjects.

**Results:**

Serum hepcidin values did not differ notably between children with viral and bacterial infection, but a significant reduction of hepcidin was noted in both groups post-infection.

**Conclusion:**

Our study provides clinical pediatric data on the role of hepcidin in the face of an acute infection. In our sample of children, hepcidin was found to rise during the acute infection and fall post-infection.

## Introduction

Hepcidin is a cytokine-induced antimicrobial peptide produced in the liver that principally regulates the homeostasis of iron concentration. Although its production can be induced by multiple stimuli, IL-6 is considered its dominant upregulator [1]. Thus hepcidin could be classified as a type II acute phase protein. The induction of hepcidin is a component of the innate immune response to infections; it decreases extracellular iron levels reducing iron availability to invading microorganisms [2].

Most studies investigate the regulation and potential roles of hepcidin in animal models [3,4]. Although hepcidin plays a key role in the development of anaemia associated with inflammation and chronic disease, there are only a few clinical studies that examine hepcidin alterations in acute or chronic infections. However, there are not sufficient data in children to validate the use of hepcidin levels in clinical algorithms for the diagnosis of acute infection.

The evaluation and treatment of children with fever without a source is a challenging and controversial clinical problem [5]. Although most well appearing children with fever have benign viral illnesses, fever might represent the first sign of occult bacteremia and subsequent serious bacterial infection [6,7]. Discrimination based on clinical criteria has not been sufficient to determine management [8].

A number of large prospective studies have established criteria to accurately identify children warranting presumptive antibiotic therapy [9,10]. Traditional laboratory screening tests include total white blood cell count, absolute neutrophil count, band to neutrophil ratio and C-reactive protein [11-13]. The use of other acute phase reactants, such as procalcitonin and IL-6, to ameliorate the sensitivity of the screen resulted in increased use of antibiotics [14-17].

During an acute-phase reaction there are dramatic changes in iron metabolism. Previous studies have demonstrated differences in serum iron parameters in children with acute bacterial versus viral infections [18]. We hypothesized that the comparison of hepcidin levels of children convalescing from bacterial and viral infections would not yield significant differences enhancing the role of hepcidin as an acute reactant protein. Thus the aim of the present study was to evaluate the alterations of serum hepcidin not only during the acute febrile phase but also post-infection.

## Materials and methods

This longitudinal study was conducted during a three-year period between 2008 and 2011. Children of Greek origin and nationality participated voluntarily in the study. Informed consent from parents was obtained in advance. The Ethics Committee of our Hospital and the University of Athens Medical School approved the research protocol.

Among 45 patients admitted to the 2nd Department of Pediatrics-University of Athens, 22 children were enrolled in the study. The subjects’ parents filled in a detailed questionnaire, with specific attention any history of anemia, chronic illness and folic acid or iron dietary supplementation. Children receiving antibiotic treatment or dietary iron supplementation as well as children with history of chronic disease or other co-morbidities were excluded from the study (18 patients). The remaining 5 febrile patients were also excluded since their infection could not be classified as viral or bacterial. All participating children underwent thorough physical examination.

The study population consisted of 22 infants and children (13 boys, mean age 28.06 ± 37.64 months, range 1 to 144 months) presenting with fever of acute onset (< 6 hours). Full blood count with differential, routine biochemical studies, C-reactive protein, hepcidin, ferritin and serum iron measurements were performed in all subjects at the time of presentation and 4 weeks later, using standard methods by the same laboratory. The hepcidin concentration samples were stored at -80°C for 5 to 10 months before analysis. Serum hepcidin-25 isoform measurements were performed by using a specific ELISA kit (DRG INTERNATIONAL Inc. 1167 U.S Highway 22 East, Mountainside, NJ 07092 USA) according to the manufacturer’s instructions [19].

Based on clinical and laboratory findings our sample was divided into two groups: the viral infection group (13 children) and the bacterial infection group (9 children). The discrimination between bacterial and viral infection was based on the combination of positive blood or urine culture along with C-reactive protein >40 mg/dl and leukocytosis with neutrophilia (white blood cell count >15.000/mm^3^, neutrophils >60%). The bacterial pathogens isolated were: *strep. pneumoniae, Esherichia Coli, Klebsiella pneumoniae, staph aureus*. The diagnosis of viral infection was based on either positive polymerase chain reaction (PCR) test or positive IgM antibodies for a specific viral agent (Epstein-Barr virus, cytomegalovirus, coxsackie virus, adenovirus). The clinical spectrum of febrile illnesses included upper or lower respiratory truct infections, urinary truct infections, occult bacteremia, gastroenteritis and cervical lymphadenitis.

### Statistical analysis

All statistical analyses and data management were performed using STATA for Windows v 8.5, (StataCorp, Texas, USA, 2006). Data are expressed as Mean ± SD, median. Our study population formed two groups, as mentioned above, the viral infection and bacterial infection groups. Each study variable was measured during the infection (variable 1) and after the infection (variable 2). Data between the two groups (variable X-viral group vs. variable X-bacterial group) were compared using non-parametric Mann-Whitney U test (independent samples). Data within each group (variable X1 vs. variable X2) were compared using non-parametric Wilcoxon paired test (paired samples). We also calculated the difference between variable 1 and variable 2 as ΔvarX = varX1-varX2 within each group and we compared these differences between the two groups (ΔvarX-viral group vs ΔvarX-bacterial group) using non-parametric Mann-Whitney U test (independent samples). P < 0.05 was considered significant.

## Results

All study variables are presented in Table 1. Significant differences are hereupon noted in detail. Serum hepcidin values were not notably different between children with viral and bacterial infection, but a significant reduction of hepcidin was noted in both groups post-infection. Serum ferritin values were similar among children belonging to the two groups; however, serum iron levels were found significantly lower only during the acute phase of bacterial infections. Within each group there were significant differences in ferritin and iron levels observed during and after infection. Significant increases in hemoglobin and hematocrit values were noted post-bacterial infection. The difference in hematocrit (Δhct) was significantly greater in the bacterial infection group as well as the difference in MCHC and reticulocyte percentages. As expected, initial ESR values were significantly higher in the bacterial infection group, resulting in a greater difference of ΕSR (ΔESR) than in the viral infection group. Similar changes were observed in WBC and neutrophil count, with higher initial values and greater differences (ΔWBC and ΔN) found in children with bacterial infections. Within each individual group initial lymphocyte count value was significantly lower, while the difference in monocyte count (ΔM) was significantly greater in the viral infection group. Initial CRP value was considerably higher in children with bacterial infections, leading to a greater difference (ΔCRP) in this group compared to the viral infection group. Differences in platelet counts within each group were significant.

**Table 1:** Characteristics of study population groups

**Δvar = var1-var2**	**Viral infection (n = 13)**	**Bacterial infection (n = 9)**	P*
Age (months)	22.8(±31.75), 8.5 (1 ~ 108)	35.66(±45.79), 18 (2 ~ 144)	
Male gender (%)	7(53.85%)	6(66.67%)	
Hepcidin1	90.52(±50.97), 79.44(11.42 ~ 210.18)	72.63(±39.95), 74.10(10.61 ~ 133.8)	0.48
Hepcidin2	53.18(±22.46), 46.17(27.12 ~ 100.73)	42.34(±28.83), 33.14(18.39 ~ 115.73)	0.12
Δhepcidin(1 → 2)	37.34(±59.89), 33.48(-89 ~ 178.74)	30.28(±38.25), 34.99(-39.44 ~ 87.61)	0.86
P**	***0.033***	***0.05***	
Ferritin1	135.98(±106.36), 68.12(30.1 ~ 333.3)	160.01(±117.97), 106.5(73.57 ~ 457.3)	0.24
Ferritin2	50.66(±37.55), 34.5(14 ~ 160)	67.65(±54.01), 39.3(12.4 ~ 173)	0.5
Δferritin(1 → 2)	85.31(±106.86), 35.58(-25.9 ~ 292.58)	92.36(±142.80), 51.7(-69 ~ 421.95)	0.71
P**	***0.0046***	***0.05***	
Serum iron1	29.69(±17.07), 28(9 ~ 69)	15.66(±4.58), 16(10 ~ 25)	***0.015***
Serum iron 2	47.69(±20.34), 46(21 ~ 76)	46.33(±27.83), 42(18 ~ 115)	0.61
ΔSerum iron (1 → 2)	-18(±29.76), -17(-67 ~ 48)	-30.66(±28.35), -24(-98 ~ 7)	0.24
P**	***0.039***	***0.01***	
Hb1(gr/dl)	11.74(±1.91), 11.7(8.1 ~ 15.5)	11.4(±1.24), 11(10.2 ~ 13.7)	0.59
Hb2 (gr/dl)	11.93(±0.92), 11.8(10.6 ~ 13.7)	12.66(±0.87), 12.1(11.7 ~ 13.9)	0.065
ΔHb(1 → 2)	-0.19(±1.63), -0.69(-2.5 ~ 3.7)	-1.26(±0.95), -1.1(-3.4 ~ -0.19)	0.17
P**	0.46	***0.007***	
Hct1 (%)	35.82(±5.62), 35.8(24.6 ~ 45.9)	34.5(±3.74), 32.9(30.3 ~ 41)	0.48
Hct2 (%)	35.30(±2.71), 35(32 ~ 40.5)	38.53(±2.48), 38.7(35.6 ~ 42)	***0.01***
Δhct(1 → 2)	0.51(±4.65), -0.70(-7.9 ~ 10.7)	-4.03(±3.02), -3.1(-10.4 ~ -1)	***0.01***
P**	0.86	***0.007***	
MCHC1	29.84(±1.58), 30(26 ~ 32.8)	29.36(±1.33), 29(27.5 ~ 31.3)	0.29
MCHC2	30.23(±1.20), 30(28 ~ 32)	30.36(±1.13), 30.2(28 ~ 32.1)	0.68
Δmchc(1 → 2)	-0.38(±1.41), -0.5(-3 ~ 2)	-1.0(±1.15), -1(-3 ~ 0.5)	0.34
P**	0.32	***0.028***	
reticulocytes1(%)	1.21(±0.66), 1(0.5 ~ 2.7)	1.6(±0.64), 1.5(0.6 ~ 2.4)	0.16
reticulocytes 2 (%)	0.86(±0.35), 0.8(0.3 ~ 1.4)	0.8(±0.36), 0.7(0.3 ~ 1.6)	0.66
Δreticulocytes (1 → 2)	0.35(±0.74), 0.3(-0.8 ~ 2.1)	0.8(±0.64), 0.6(0–1.7)	0.13
P**	0.10	***0.012***	
ESR1 (mm/1 h)	17.38(±8.11), 15(5 ~ 31)	37.66(±14.04), 38(19 ~ 70)	***0.0007***
ESR2 (mm/1 h)	11.38(±8.7), 9(4 ~ 35)	12.44(±8.5), 10(4 ~ 30)	0.68
ΔESR(1 → 2)	6.0(±12.32), 3(-15 ~ 26)	25.22(±12.36), 26(8 ~ 49)	***0.0033***
P**	0.1	***0.007***	
WBC1/mm^3^	10205(±3806), 10100(4200 ~ 18200)	23133(±13813), 17400(9300 ~ 50100)	***0.005***
WBC2/mm^3^	10100(±3003), 8900(6000 ~ 16800)	10855(±5008), 10400(4700 ~ 21100)	0.86
Δwbc(1 → 2)	115(±4306), 200(-6300 ~ 9700)	12277(±12920), 7700(-4000 ~ 34100)	***0.016***
P**	0.94	***0.02***	
N1 (%)	47.38(±24.59), 54(11 ~ 89)	69.55(±16.43), 66(40 ~ 89)	***0.02***
N2 (%)	37.07(±11.62), 35(21 ~ 63)	37.17(±15.87), 41(10 ~ 55)	0.73
ΔN(1 → 2)	10.3(±18.19), 16(-24 ~ 33)	37.37(±18.42), 37(-1 ~ 56)	***0.01***
P**	0.063	***0.0109***	
L1 (%)	37.3(±23.08), 33(5 ~ 72)	19.88(±10.44), 23(6 ~ 33)	0.08
L2 (%)	49.61(±14.20), 52(27 ~ 72)	48.63(±18.18), 45(32 ~ 82.5)	0.78
ΔL(1 → 2)	-12.3(±18.71), -17(-39 ~ 24)	-28.74(±16.85), -28(-55.5 ~ -7)	0.065
P**	***0.042***	***0.007***	
M1 (%)	12.38(±5.79), 12(6 ~ 24)	8.66(±5.52), 6(3 ~ 20)	0.069
M2 (%)	8.23(±2.80), 7(4 ~ 13)	10.03(±3.45), 10(6 ~ 16)	0.19
ΔM(1 → 2)	4.15(±6.4), 2(-4 ~ 18)	-1.36(±6.39), -3(-10 ~ 10)	***0.034***
P**	0.072	0.4	
CRP1 (mg/dl)	17.07(±24.69), 12(0 ~ 93)	100.88(±59.50), 92(24 ~ 178)	***0.0006***
CRP2 (mg/dl)	4.57(±5.48), 1(0 ~ 17)	5.11(±9.51), 2(0 ~ 30)	0.91
Δcrp(1 → 2)	12.5(±23.08), 7(-9 ~ 82)	95.77(±64.87), 90.5(-6 ~ 173)	***0.0037***
P**	***0.022***	***0.0109***	
PLT1 /mm^3^	382923(±162607), 335000(191000 ~ 758000)	465444(±162687), 438000(215000 ~ 709000)	0.19
PLT2 /mm^3^	275538(±63602), 278000(198000 ~ 403000)	302555(±48844), 303000(230000 ~ 383000)	0.25
ΔPLT(1 → 2)	107384(±137673), 44000(-84000 ~ 355000)	162888(±132197), 120000(-41000 ~ 326000)	0.3
P**	***0.015***	***0.0109***	

P* Mann-Whitney U test.

P** Wilcoxon paired test.

Data are expressed as mean (±SD), median.

## Discussion

Our study provides clinical pediatric data on the role of hepcidin in the face of an acute infection. In our sample of children with viral and bacterial infections, hepcidin was found to rise during the acute infection and fall post-infection.

Hepcidin is a 25-amino acid peptide synthesized by hepatocytes, secreted into the bloodstream and cleared from the circulation by the kidneys. It regulates the levels of circulating iron by controlling the absorption of dietary iron and the release of iron from macrophages and hepatocytes. Hepcidin synthesis is regulated by both physiologic and pathologic mechanisms. In peripheral blood leads in decrease in hepcidin concentration In hypoxia, iron deficiency, anemia or ineffective erythropoiesis, increased iron demand in the peripheral blood leads to a decrease in hepcidin concentation. The latter results in increased absorption of iron from the intestine and increased release from iron-storing cells by binding, internalization and degradation of the cellular iron efflux molecule, ferroportin [20].

On the contrary, infection, chronic inflammation and iron overload result in increases of hepcidin concentration and subsequent decreases in circulating iron; in hereditary hemochromatosis a mutated hepcidin gene results in deficient excretion of the molecule leading to iron accumulation.

Most studies examining the role of hepcidin as an acute phase protein are based on in vitro and animal models. Specifically, hepcidin has been well described as a hepatic acute phase protein in laboratory studies similarly to ferritin. Ferritin rises during acute infections resulting in unreliable measurements as an iron parameter. Murine models indicate that hepcidin is a component of the innate immune response to acute infection with anti-inflammatory properties and a potential marker of billiary stress [21,22]. Experimental deliberate exposure to bacterial agents in salmon and rats has demonstrated the up regulation of hepcidin [23,24].

Limited research data from adult populations attempt to elucidate the role of hepcidin in the common cold, iron metabolism disorders, chronic kidney disease, chronic hepatitis C and kidney transplant recipients [25-27]. In an adult patient population study group, hepcidin was demonstrated as a potentially useful marker that reflects the degree of billiary inflammation in cholecystitis and primary sclerosing cholangitis (PSC) - associated bacterial infection. To the best of our knowledge, there are only two studies examining the role of serum hepcidin during chronic infections in pediatric populations. One showed a correlation of increased hepcidin levels with asymptomatic malarial parasitemia (P. falciparum and P. vivax), despite the absence of a measurable acute phase response [28]. The second failed to demonstrate significant alterations of hepcidin levels in children with chronic gastrointestinal infections (*H. pylori* and helminthes) [29].

The main limitation of our study was the small size of sample. However, since weak statistical significance was achieved, a larger sample is needed to clarify the changes in hepcidin levels. The comparison of its levels in children convalescing from bacterial and viral infections did not yield significant differences. Interestingly, low levels of serum iron were shown in bacterial infections. These findings generate thoughts about the existence of other biological parameters involved in the regulation of iron metabolism during acute infection.

## Conclusions

The present study provides preliminary data on the role of hepcidin as an acute reaction marker in febrile children. Further studies are needed to clarify our findings, ideally in a larger sample of febrile children. In addition other biological parameters involved in iron metabolism could be investigated.

## Abbreviations

Hb: Hemoglobin; Ht: Hematocrit; MCHC: Mean corposcular hemoglobin concentration; ESR: Erythrocyte sedimentation rate; WBC: White blood cells; N: Neutrophils; L: Lymphocytes; M: Monocytes; CRP: C-reactive protein; PLT: Platelets.

## Competing interests

The authors declare that they have no competing interests.

## Authors’ contributions

LK: design the study and enroll the subjects. AS: participated in the selection of the subjects. DG: measured the serum hepcidin samples. LS: measured the serum hepcidin samples. CT: did the statistical analysis. All authors read and approved the final manuscript.

## References

[B1] Bode JG Albrecht U Häussinger D Heinrich PC Schaper F Hepatic acute phase proteins-Regulation by IL-6- and IL-1-type cytokines involving STAT3 and its crosstalk with NF-κB-dependent signaling Eur J Cell Biol 2012 91 6–7 496 506 22093287 10.1016/j.ejcb.2011.09.008

[B2] Ganz T Nemeth E Hepcidin and disorders of iron metabolism Annu Rev Med 2011 62 347 360 10.1146/annurev-med-050109-142444 20887198

[B3] Armitage AE Eddowes LA Gileadi U Cole S Spottiswoode N Selvakumar TA Ho LP Townsend AR Drakesmith H Hepcidin regulation by innate immune and infectious stimuli Blood 2011 118 15 4129 4139 10.1182/blood-2011-04-351957 21873546

[B4] Pagani A Nai A Corna G Bosurgi L Rovere-Querini P Camaschella C Silvestri L Low hepcidin accounts for the proinflammatory status associated with iron deficiency Blood 2011 118 3 736 746 10.1182/blood-2011-02-337212 21628413

[B5] Chancey RJ Jhaveri R Fever without localizing signs in children: a review in the post-Hib and postpneumococcal era Minerva Pediatr 2009 61 5 489 501 19794375

[B6] Mintegi S Benito J Sanchez J Azkunaga B Iturralde I Garcia S Predictors of occult bacteremia in young febrile children in the era of heptavalent pneumococcal conjugated vaccine Eur J Emerg Med 2009 16 4 199 205 10.1097/MEJ.0b013e32831cefc9 19593900

[B7] Wilkinson M Bulloch B Smith M Prevalence of occult bacteremia in children aged 3 to 36 months presenting to the emergency department with fever in the postpneumococcal conjugate vaccine era Acad Emerg Med 2009 16 3 220 225 10.1111/j.1553-2712.2008.00328.x 19133844

[B8] Baraff LJ Management of infants and young children with fever without source Pediatr Ann 2008 37 10 673 679 10.3928/00904481-20081001-01 18972849

[B9] Pantell RH Newman TB Bernzweig J Bergman DA Takayama JI Segal M Finch SA Richard MA Wasserman C Management and outcomes of care of fever in early infancy JAMA 2004 291 1203 1212 10.1001/jama.291.10.1203 15010441

[B10] Seow VK Lin AC Lin IY Chen CC Chen KC Wang TL Chong CF Comparing different patterns for managing febrile children in the ED between emergency and pediatric physicians: impact on patient outcome Am J Emerg Med 2007 25 9 1004 1008 10.1016/j.ajem.2007.03.001 18022493

[B11] Furer V Raveh D Picard E Goldberg S Izbicki G Absence of leukocytosis in bacteraemic pneumococcal pneumonia Prim Care Respir J 2011 20 3 276 281 10.4104/pcrj.2011.00023 21509416 PMC6549842

[B12] Isaacman DJ Burke BL MS utility of the serum c-reactive protein for detection of occult bacterial infection in children Arch Pediatr Adolesc Med 2002 156 905 909 12197798 10.1001/archpedi.156.9.905

[B13] Pulliam PN Attia MW Cronan KM C-reactive protein in febrile children 1 to 36 months of age with clinically undetectable serious bacterial infection Pediatrics 2001 108 6 1275 1279 10.1542/peds.108.6.1275 11731648

[B14] Strait RT Kelly KJ Kurup VP Tumor necrosis factor-alpha, interleukin-1 beta, and interleukin-6 levels in febrile, young children with and without occult bacteremia Pediatrics 1999 104 6 1321 1326 10.1542/peds.104.6.1321 10585983

[B15] Thayyil S Shenoy M Hamaluba M Gupta A Frater J Verber IG Is procalcitonin useful in early diagnosis of serious bacterial infections in children? Acta Paediatr 2005 94 2 155 158 10.1080/08035250410025140 15981747

[B16] Guen CG Delmas C Launay E Caillon J Loubersac V Picherot G Roze JC Contribution of procalcitonin to occult bacteraemia detection in children Scand J Infect Dis 2007 39 2 157 159 10.1080/00365540600904753 17366034

[B17] Manzano S Bailey B Girodias JB Galetto-Lacour A Cousineau J Delvin E Impact of procalcitonin on the management of children aged 1 to 36 months presenting with fever without source: a randomized controlled trial Am J Emerg Med 2010 28 6 647 653 10.1016/j.ajem.2009.02.022 20637377

[B18] Kossiva L Gourgiotis DI Tsentidis C Anastasiou T Maramarinos A Vasilenko H Sdogou T Georgouli H Serum hepcidin and ferritin to iron ratio in evaluation of bacterial versus viral infections in children: a single-center study Pediatr Infect Dis J 2012 31 8 795 798 10.1097/INF.0b013e318256f843 22531233

[B19] Geerts I Vermeersch P Joosten E Evaluation of the first commercial hepcidin ELISA for the differential diagnosis of anemia of chronic disease and iron deficiency anemia in hospiatized geriatric patients ISRN Hematol 2012 2012 567491 Epub 2012 Feb 29 10.5402/2012/567491 22461996 PMC3302103

[B20] Eleftheriadis T Liakopoulos V Antoniadi G Kartsios C Stefanidis I The role of hepcidin in iron homeostasis and anemia in hemodialysis patients Semin Dial 2009 22 1 70 77 10.1111/j.1525-139X.2008.00532.x 19250447

[B21] Motley ST Morrow BJ Liu X Dodge IL Vitiello A Ward CK Shaw KJ Simultaneous analysis of host and pathogen interactions during an in vivo infection reveals local induction of host acute phase response proteins, a novel bacterial stress response, and evidence of a host-imposed metal ion limited environment Cell Microbiol 2004 6 9 849 865 10.1111/j.1462-5822.2004.00407.x 15272866

[B22] Strnad P Schwarz P Rasenack MC Kucukoglu O Habib RI Heuberger D Ehehalt R Müller MW Stiehl A Adler G Kulaksiz H Hepcidin is an antibacterial, stress-inducible peptide of the biliary system PLoS One 2011 6 1 e16454 10.1371/journal.pone.0016454 21283681 PMC3025980

[B23] Martin SA Blaney SC Houlihan DF Secombes CJ Transcriptome response following administration of a live bacterial vaccine in Atlantic salmon (Salmo salar) Mol Immunol 2006 43 11 1900 1911 10.1016/j.molimm.2005.10.007 16313960

[B24] Malik IA Naz N Sheikh N Khan S Moriconi M Blaschke M Ramadori G Comparison of changes in gene expression of transferrin receptor-1 and other iron-regulatory proteins in rat liver and brain during acute-phase response Cell Tissue Res 2011 344 299 312 10.1007/s00441-011-1152-3 21437659 PMC3085758

[B25] Hoppe M Hulthén L Capturing the onset of the common cold and its effects on iron absorption Eur J Clin Nutr 2007 61 8 1032 1034 10.1038/sj.ejcn.1602614 17268421

[B26] Kemna EH Kartikasari AE van Tits LJ Pickkers P Tjalsma H Swinkels DW Regulation of hepcidin: insights from biochemical analyses on human serum samples Blood Cells Mol Dis 2008 40 3 339 346 10.1016/j.bcmd.2007.10.002 18023212

[B27] Malyszko J Malyszko JS Pawlak K Mysliwiec M Hepcidin, an acute-phase protein and a marker of inflammation in kidney transplant recipients with and without coronary artery disease Transplant Proc 2006 38 9 2895 2898 10.1016/j.transproceed.2006.08.137 17112858

[B28] de Mast Q Syafruddin D Keijmel S Riekerink TO Deky O Asih PB Swinkels DW van der Ven JA Increased serum hepcidin and alterations in blood iron parameters associated with asymptomatic P. falciparum and P. vivax malaria Haematologica 2010 95 1068 1074 10.3324/haematol.2009.019331 20133896 PMC2895029

[B29] Cherian S Forbes DA Cook AG Sanfilippo FM Kemma EH Swinkels DW Burgner DP An insight into the relationships between hepcidin, anemia, infections and inflammatory cytokines in pediatric refugees: a cross-sectional study PLoS One 2008 3 12 e4030 10.1371/journal.pone.0004030 19107209 PMC2603326

